# Brain Abscess Complicating Metastatic Scalp Basal Cell Carcinoma With a Malignant Fungating Wound: A Case Report and Literature Review

**DOI:** 10.7759/cureus.88592

**Published:** 2025-07-23

**Authors:** Sophia Ma, Tarek Zieneldien, Janice Kim, John Greene

**Affiliations:** 1 School of Medicine, Johns Hopkins University, Baltimore, USA; 2 Dermatology, Johns Hopkins University School of Medicine, Baltimore, USA; 3 College of Arts and Sciences, Michigan State University College of Osteopathic Medicine, East Lansing, USA; 4 Internal Medicine, Moffitt Cancer Center, Tampa, USA

**Keywords:** basal cell carcinoma, brain abscesses, malignant fungating wounds, methicillin-sensitive staphylococcus aureus (mssa), palliative care

## Abstract

Basal cell carcinoma (BCC), the most prevalent skin cancer, rarely metastasizes to the brain. Here, we report a case of a 63-year-old man with metastatic BCC complicated by a malignant fungating wound (MFW), a cancerous lesion seen in advanced diseases, and *Staphylococcus aureus* infection, resulting in brain abscesses and encephalopathy. The BCC initially involved the scalp and was treated with excision but later recurred and metastasized. It was associated with a chronic, open MFW lesion with necrosis and ulceration. MRIs revealed cranial structure involvement and brain abscesses. Despite treatment efforts, the patient’s condition deteriorated, manifesting cognitive symptoms, ultimately leading to his placement in palliative care. Based on the findings of this case, vigilant monitoring, wound culturing of non-healing or necrotic, high-risk lesions to detect infections earlier, and appropriate palliative measures to manage MFWs may help improve patient outcomes.

## Introduction

Basal cell carcinoma (BCC) is the most common type of skin cancer, arising from epidermal basal cells. Although it is locally aggressive if left untreated, BCC rarely metastasizes, with histologically confirmed reported metastasis incidence rates ranging from 0.0028% to 0.55% [1]. BCC primarily affects adults, particularly the elderly, but in recent years its incidence has been increasing in adults under 50 years of age [1].

While cases of untreated or recurrent scalp BCC invading cranial structures have been reported, the occurrence of brain abscesses, devastating infections that may arise from tumor necrosis, immunosuppression (e.g., steroid therapy), or secondary infection from chronic wounds is rare in BCC [2].

In advanced stages, BCC can develop into ulcerative, necrotic lesions known as malignant fungating wounds (MFWs), which affect approximately 5-15% of patients with metastatic cancer [3,4]. MFWs arise from cancerous infiltration of the skin, leading to tissue hypoxia and inflammation [4]. The consequent purulence and bleeding create an ideal environment for microbial invasion, commonly bacteria such as *Staphylococcus, Pseudomonas, Streptococcus, Enterococcus, Proteus, Escherichia, *and* Corynebacterium* [3]. The management of MFWs typically requires a multidisciplinary approach, with treatment focusing on symptom control, infection management, and palliative care rather than wound healing, as these wounds are generally highly susceptible to infections, incurable, and associated with end-stage disease [3,4]. This case report highlights a rare and complex manifestation of BCC-associated MFW and brain abscesses, underscoring the need for further exploration of such severe complications and emphasizing the importance of early detection and management of advanced BCC.

## Case presentation

**Figure 1:**
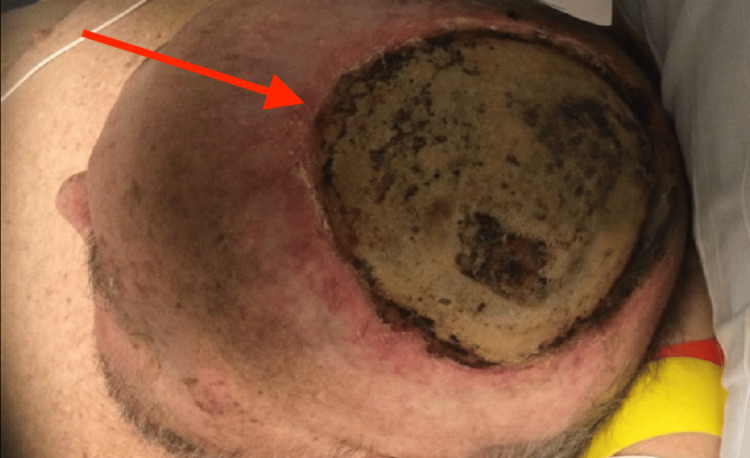
BCC involving the scalp, presenting as a large, ulcerated, malignant fungating wound with underlying bone invasion. BCC: Basal cell carcinoma

This case describes a 63-year-old man diagnosed with stage IV metastatic BCC involving the scalp and postauricular area. The cancer was associated with a chronic, open, left-sided lesion that had developed into an MFW, characterized by rapid growth, necrosis, and ulceration. The MFW was accompanied by symptoms such as pain, bleeding, and infection, significantly impacting the patient's quality of life. The disease had metastasized to the bone and brain, resulting in brain abscesses (Figure 1).

**Figure 2:**
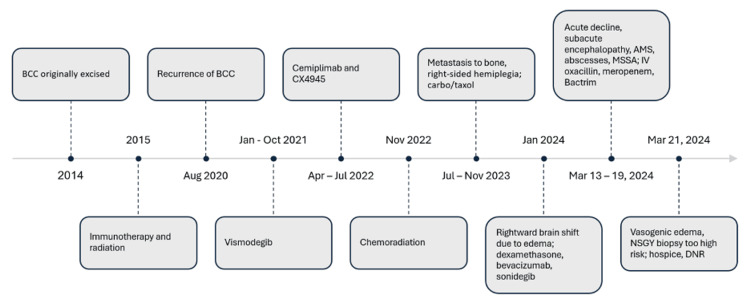
Timeline of disease and treatment course. BCC: Basal cell carcinoma; MSSA: methicillin-susceptible *Staphylococcus aureus*; DNR: do not resuscitate;AMS: altered mental status

The BCC of the scalp and head was originally treated with excision in 2014, followed by immunotherapy and radiation in 2015. However, in August 2020, a biopsy confirmed recurrence involving the left superior frontal scalp, right superior parietal scalp, and right inferior postauricular area. The patient was treated with vismodegib, cemiplimab, oral CX4945 drug, and chemoradiation therapy. The patient was additionally treated with carboplatin/paclitaxel chemotherapy from July 2023 to November 2023 due to biopsy-proven metastatic disease to bone (Figure 2).

Contralateral hemiplegia in left upper and lower extremities due to cognitive changes began progressively after the carboplatin/paclitaxel infusion. In November 2023, the patient experienced generalized headaches, extreme pain and bleeding at the scalp where the BCC was excised. In January 2024, MRIs depicted a 4 mm rightward midline shift due to a large area of edema in the left frontoparietal lobe surrounding an enhancing lesion in the mesial frontoparietal area near the vertex, which corresponded to an FDG avid lesion. The patient was started on bevacizumab and sonidegib, which eventually led to improvement of the right-sided weakness. 

Comorbidities recorded upon admission in January 2024 included melanoma, BCC, cutaneous squamous cell carcinoma, leukocytosis due to steroid therapy, neoplasm-related pain, and secondary anemia of chronic disease. The patient was also undergoing therapy and wound care for his open scalp and posterolateral right neck wounds. Despite aggressive management with tramadol, Avastin, intravenous hydromorphone, and high-dose dexamethasone, the disease continued to progress. 

**Figure 3:**
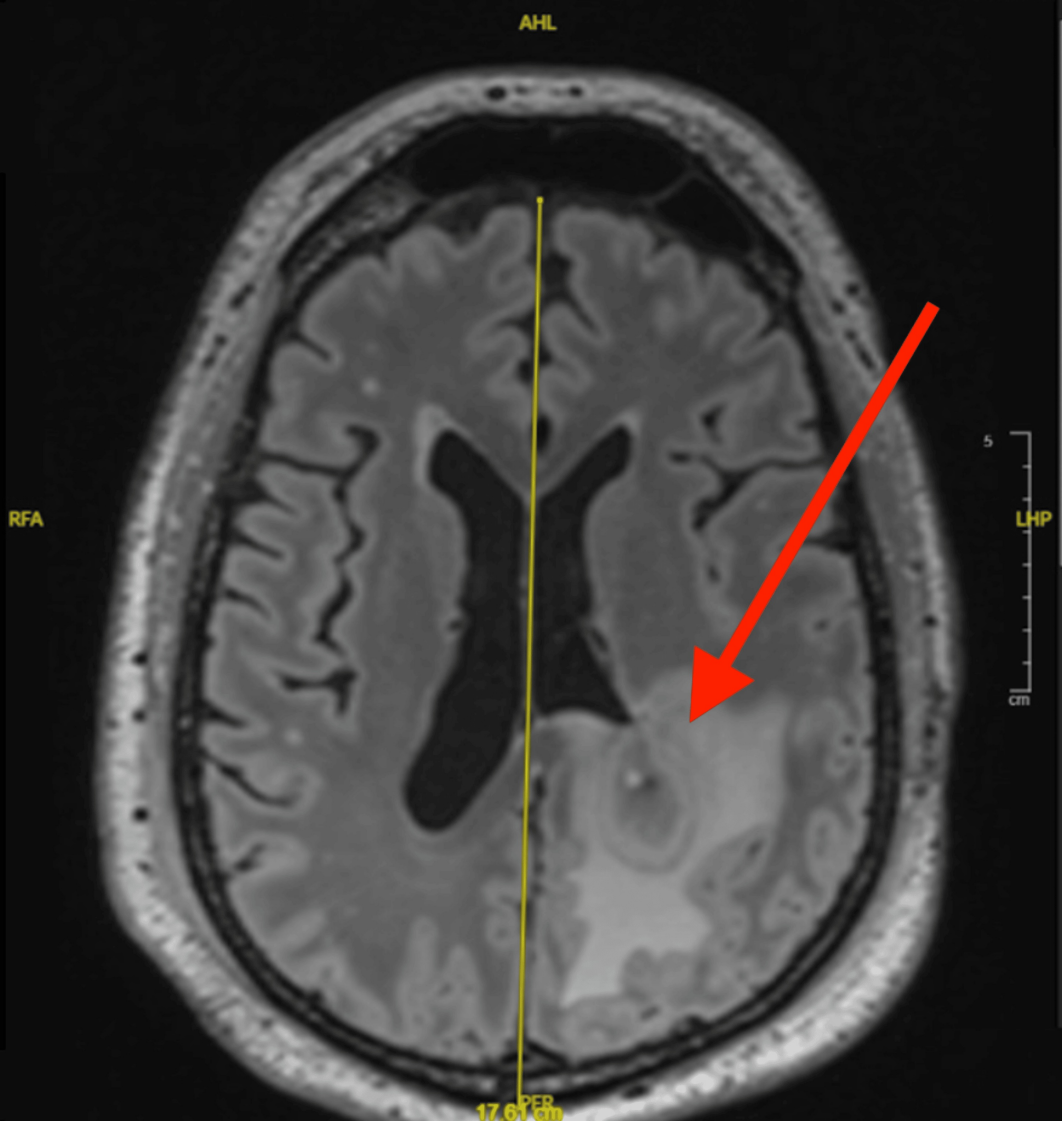
MRI with and without contrast: Axial 3D multiplanar reconstruction (MPR) FLAIR image depicting a well-defined, peripherally enhancing lesion in the left parietal subcortical white matter inferior to the resection site. The lesion shows marked restricted diffusion centrally, compatible with an abscess. MRI study dated March 13, 2024. FLAIR: Fluid-attenuated inversion recovery

**Figure 4:**
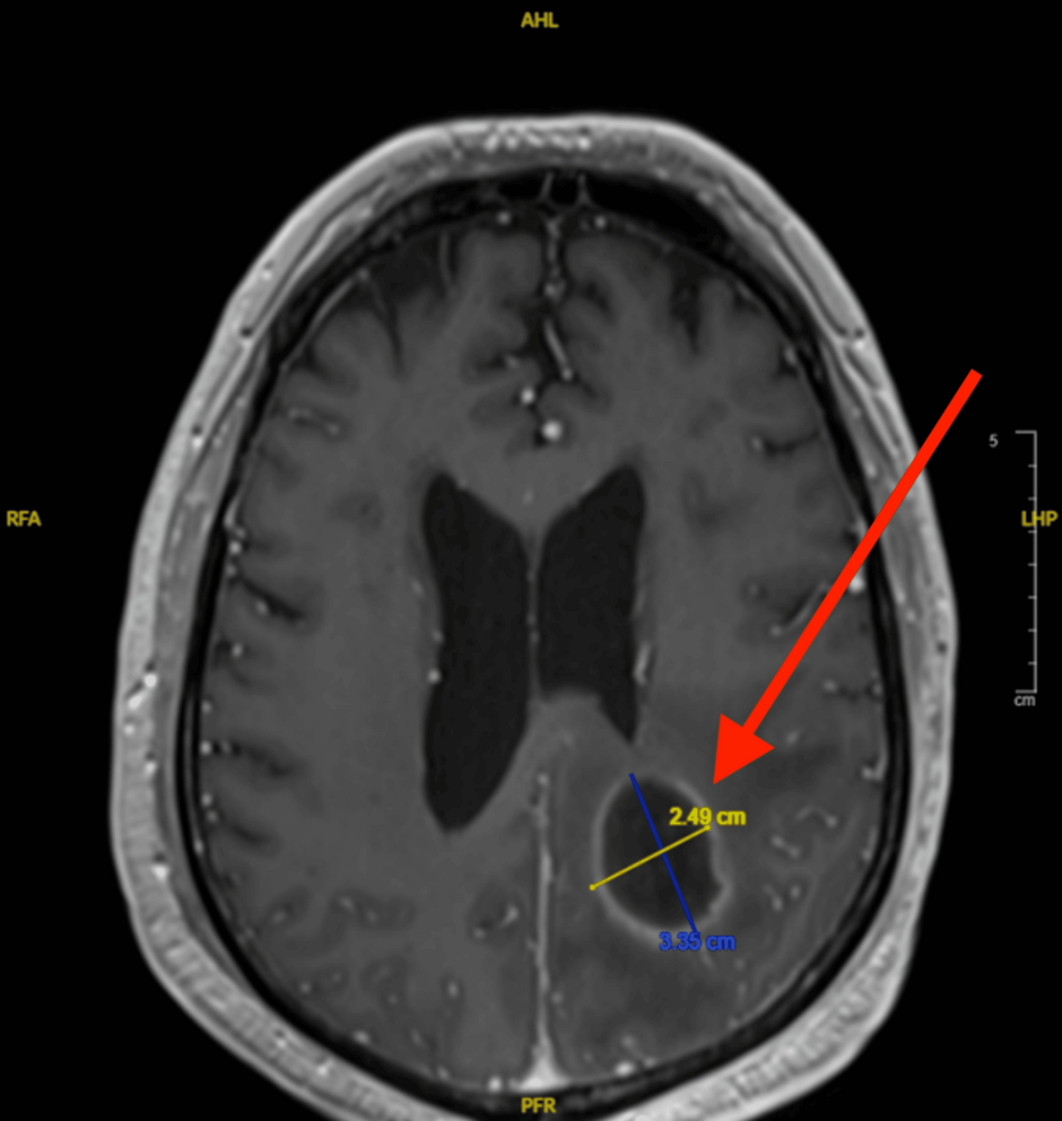
Contrast enhanced 3-D axial T1-weighted magnetization prepared rapid gradient echo (MPRAGE) MPR image depicting a 2.5 x 3.4 cm left parietal periventricular abscess. MRI study dated March 13, 2024. MPR: Multiplanar reconstruction

**Figure 5:**
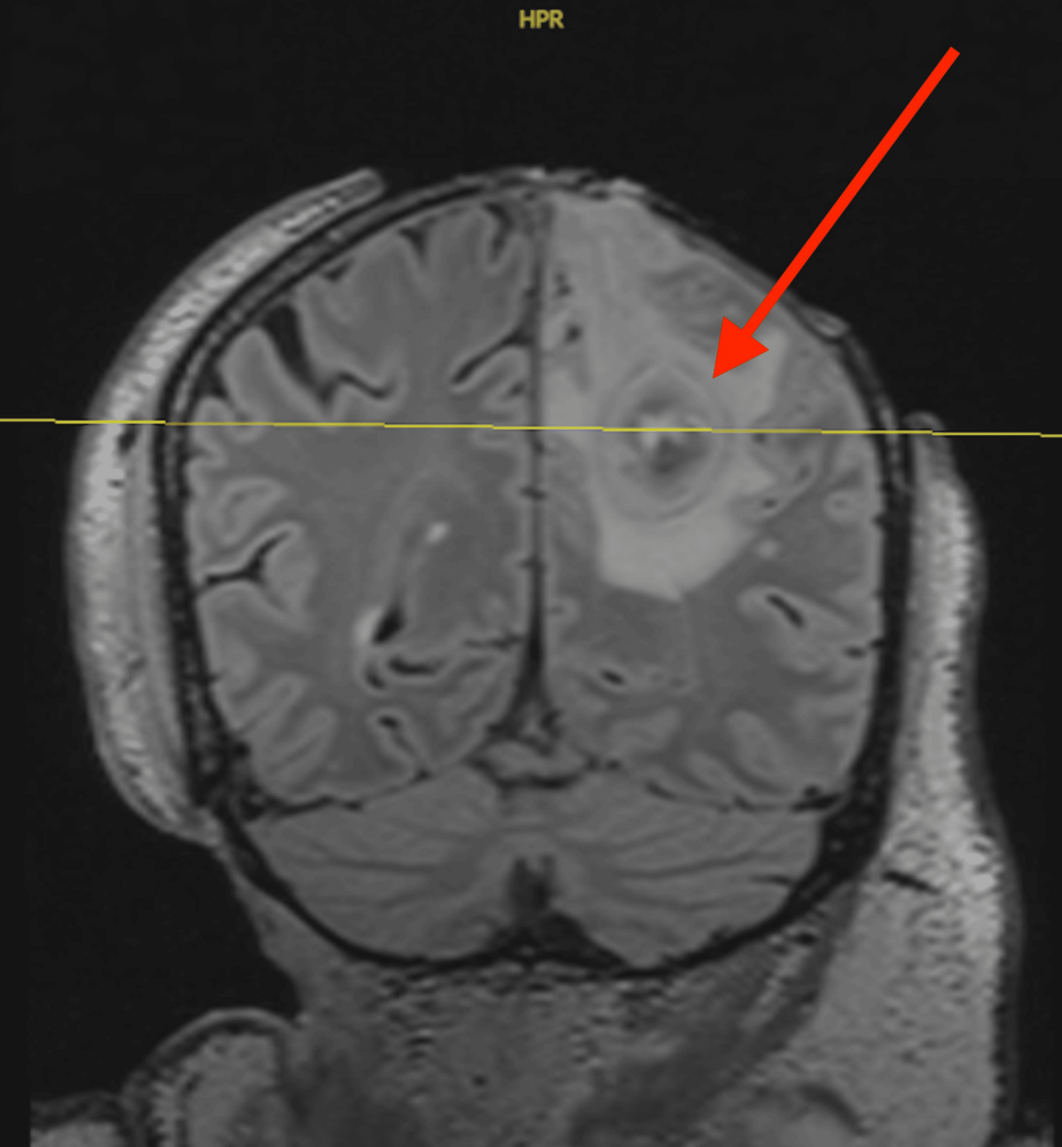
MRI Brain with and without contrast: Coronal FLAIR 3D MPR reformat image depicting large scalp defects in the frontoparietal vertex and right occipital regions, with irregularity of the underlying bone along the outer table. There are small areas of apparent loss of integrity in the inner table of the left parietal bone, as well as pus in the surgical resection cavity, located superior to the lesion described above, distributed across three different pockets. MRI study dated March 13, 2024. FLAIR: Fluid-attenuated inversion recovery; MPR: multiplanar reconstruction

In March 2024, the patient presented with agitation, altered mental status, intermittent confusion, difficulty with speech, worsening weakness and headache, and subacute encephalopathy due to the large parietal abscess. His mentation waxed and waned, and his condition continued to decline. The patient’s mental status changes preceded radiographic detection of abscesses, raising concern for early intracranial infection and leading to same-day MRI evaluation. Imaging revealed four pockets of large, left-sided, acute, parietal brain abscesses with surrounding vasogenic edema and tumor necrosis that were suspected to be polymicrobial (Figure 3). The largest abscess was inferior to the resection site in the left parietal periventricular region and measured 2.5 cm x 3.4 cm; the other three abscesses conformed to​​ the prior resection site (Figure 4). Imaging also revealed a large scalp defect in the frontoparietal vertex and right occipital regions with irregularity of the underlying bone along the outer table (Figure 5). PET scans showed a moth-eaten appearance of the left frontal calvarium with areas of associated fluorodeoxyglucose uptake near the vertex. Additionally, a cyst-like density previously observed on an MRI from January 2024 showed increased focus. 

The superficial scalp wound culture grew gram-positive methicillin-susceptible *Staphylococcus aureus* (MSSA) that was resistant to penicillin, clindamycin, and erythromycin; intermediate to gentamicin; and susceptible to linezolid, oxacillin, Synercid, rifampin, tetracycline, tigecycline, trimethoprim/sulfamethoxazole, and vancomycin. In addition, a Karius blood test that picks up over 1,300 pathogens was positive for MSSA. The CNS infection was managed with a combination of oxacillin (2,000 mg, IVPB, Q4HR) and meropenem (1,000 mg, IVPB, Q6HR) to provide empiric, broad-spectrum coverage against anaerobic and gram-negative organisms, given the high risk of a polymicrobial brain abscess. This regimen also offered advantages including enhanced CNS penetration and potential for synergistic therapeutic effects. Bactrim (trimethoprim/sulfamethoxazole) was also administered for *Pneumocystis jirovecii* pneumonia prophylaxis while he was on steroids. Although he had previously had multiple resections, the patient was deemed too high risk for biopsy of his brain abscesses due to significant neurosurgical risks, including multifocal brain abscesses, poor performance status as evidenced by progressive neurological decline, and high risk for recurrent infections given his open wound. 

After further developing acute metabolic encephalopathy, hyponatremia, acute intractable headache, and acute chronic right hemiplegia, hospice comfort measures focusing on pain and symptom management were deemed most appropriate given the patient’s end-stage condition. Treatment included intravenous antibiotics for six weeks and enteral nutrition with long-term acute care.

## Discussion

Progressive, refractory BCC, including metastatic and locally advanced forms, often occurs in high-risk anatomical regions such as the neck, eyes, head, nose, and ears [5]. Despite its typical slow growth and local invasiveness, this case demonstrates its potential for extensive metastasis to the bone and brain, which is exceedingly uncommon [6]. While there have previously been reports of intracranial abscesses secondary to scalp BCC invasion, to our knowledge, this is the first reported case of the occurrence of multifocal discrete parenchymal brain abscesses arising in the context of recurrent, metastatic scalp BCC with an MFW, despite oncologic and antimicrobial therapy. This differs from the more typical scenario, where BCC directly invades the intradural space and presents as a tumor extending into the brain [2]. The unusual progression in this patient’s case likely reflects the combined effects of a chronic, recurrent MFW promoting tumor resistance and aggressiveness, alongside immunosuppression from multiple prior skin cancers, extensive oncologic treatments, and chronic steroid use, which together facilitated both tumor progression and severe infectious complications.

The patient was initially treated with surgical excision, the standard treatment for BCCs, then inhibitors, immunotherapy, chemotherapy, and radiation therapy [7]. However, the development of resistance over time remains challenging in managing metastatic BCC involving the brain, as it can lead to progressive, non-healing MFWs [8]. These chronic wounds, particularly in immunocompromised patients, create an environment susceptible to severe infectious complications such as brain abscesses, as depicted in this case [3]. In this patient, the diagnosis of abscesses was distinguished from tumor recurrence or radiation necrosis by the presence of rim-enhancing, diffusion-restricted lesions on MRI, clinical evidence of infection, positive MSSA wound cultures, and a positive MSSA Karius blood test. Additionally, the chronic MSSA-colonized scalp lesion and calvarial irregularity suggest direct spread, likely through osteomyelitis, with possible hematogenous seeding supported by the positive MSSA Karius blood test and the patient’s immunosuppression from steroids and hedgehog inhibitors.

Furthermore, in the treatment of advanced head and neck cancer patients with MFWs, given the need to manage pain, odor, and infection, palliative care is frequently prioritized since it can improve patient quality of life, symptom control, and lessen the cost of care [9]. These wounds, resulting from tumor infiltration, poor vascularization, and necrosis, provide an optimal microenvironment for microbial growth and increase the risk for life-threatening infections [3,10]. In our case, the unresectable scalp lesion with calvarial involvement was colonized and infected by MSSA, as confirmed by wound cultures and the Karius blood test. This underscores the importance of regular wound swab cultures and antibiotic susceptibility testing, particularly in immunocompromised patients, to both distinguish infection-related tissue necrosis from hypoperfused tumor progression and guide appropriate treatment. In this case, neurosurgical drainage was deemed inappropriate due to the patient’s multifocal abscesses, poor functional status, chronic immunosuppression due to steroid use, and high procedural risk. When surgical or barrier control is not possible, symptom-focused wound care, including appropriate antibiotics and antiseptic dressings such as iodopovidone-impregnated or silver dressings, remains the standard management for unresectable MFWs of the scalp and skull, prioritizing goals of reducing odor, controlling infection, and improving quality of life [11,12].

## Conclusions

Further research into SMO/GLI-targeted combination therapies and immune checkpoint modulation in advanced BCC may help improve patient outcomes, as persistent, treatment-refractory disease with skull invasion can lead to chronic wounds that are highly susceptible to life-threatening infections such as MSSA brain abscesses, especially in immunosuppressed patients. Moreover, the progression from a chronic open scalp lesion to aggressive brain metastases suggests that early detection and close monitoring are important in managing high-risk BCC lesions involving cranial structures. Additionally, further studies should explore the development of standardized multidisciplinary protocols for managing MFWs, including wound care and infection prevention strategies. Consideration of practical surveillance strategies for high-risk or locally advanced BCC may include contrast-enhanced brain MRI every 3-6 months to monitor for potential skull or brain invasion, particularly in cases with concerning clinical features. This case contributes to the limited literature on metastatic BCC-associated MFWs and brain abscesses, highlighting the need for increased awareness of such rare but severe complications.
